# Risk-stratified patients with resectable soft tissue sarcoma benefit from epirubicin-based adjuvant chemotherapy

**DOI:** 10.1002/cam4.209

**Published:** 2014-02-27

**Authors:** Aaron D Schenone, Jingqin Luo, Luke Montgomery, Daniel Morgensztern, Douglas R Adkins, Brian A Van Tine

**Affiliations:** 1 Division of Medical Oncology, Washington University in St. LouisSt. Louis, 63110, Missouri; 2 College of Medicine, Des Moines UniversityDes Moines, 50312, Iowa; 3 Division of Biostatistics, Washington University in St. LouisSt. Louis, 63110, Missouri; 4 Siteman Cancer Center, Washington University in St. LouisSt. Louis, 63110, Missouri; 5 Loyola UniversityChicago, 60626, Illinois

**Keywords:** Adjuvant chemotherapy, doxorubicin, epirubicin, sarcoma

## Abstract

As adjuvant chemotherapy (AC) for soft tissue sarcomas is controversial, we performed a retrospective analysis of patients seen at Washington University in St. Louis to evaluate whether it benefited our patient population. Patients were risk-assessed using the Memorial Sloan Kettering Predictive Nomogram (MSKPN). We defined high-risk patients by a MSKPN 4-year postoperative probability of sarcoma-specific death of ≥0.3 and investigated if they benefited from AC. Retrospective review was performed on patients seen between 15 February 1996 and 6 February 2010. A propensity score method in the logistic regression framework was used to model the likelihood of receiving AC. To make causal inference on the effect of AC on survival outcomes, a propensity score inverse probability of treatment weighting approach was applied to survival analysis. Overall, 135 high-grade patients were assessed, 33 were treated with Ifosfamide/Epirubicin (I/Epi) and 102 were non AC patients. The stratified MSKPN risk was not significantly associated with any survival endpoint in the whole cohort, but trended for overall survival (OS) when evaluated against non AC patients. After adjustment for MSKPN risk and other variables, patients not receiving chemotherapy had significantly worse OS, recurrent free survival, and disease-specific survival (DSS) with adjusted hazard ratios of 4.18 (95% CI: 2.22–7.90), 8.96 (95% CI: 3.85–20.83), and 5.42 (95% CI: 2.09–14.06), respectively. In retrospective analyses, risk-stratified patients with soft tissue sarcoma benefited from I/Epi-based AC. Randomized I/Epi versus I/Doxorubicin clinical trials may determine the optimal adjuvant treatment.

## Introduction

Soft tissue sarcomas (STS) are cancers of mesenchymal origin with an US annual incidence of approximately 11,000 per year 1. The treatment of nonmetastatic sarcoma includes en bloc resection with or without radiation depending on grade, histology, size, and margin status 2. When combined with adjuvant radiotherapy, extremity STS have shown improved 5-year disease-free survival (DFS) up to 22% 3,4. Due to molecular heterogeneity, the role of neoadjuvant and adjuvant chemotherapy (AC) remains controversial, albeit commonly used by some institutions as standard of care.

A Cochrane systematic review of 14 clinical trials including all primary sites confirmed reduction in recurrence, improved DFS, and trend toward OS for anthracycline-based AC groups 5. A second systematic meta-analysis of 17 randomized controlled trials of patients receiving AC had recurrent free survival (RFS) and OS benefit found to be greatest within combination Ifosfamide/Epirubicin (I/Epi) trials 6. The most compelling trial for the use of AC to prevent recurrence was by Italian Sarcoma Study Group, which demonstrated a 5-year OS probability of 0.66 versus 0.46 for adjuvant I/Epi and control groups, respectively 7. A follow-up phase III trial using three cycles of neoadjuvant I/Epi with or without two additional postoperative cycles saw benefit with an epirubicin-based regimen demonstrated a 5-year OS probability of 0.70 8. Based on these data, epirubicin replaced doxorubicin as standard of care for AC at Washington University

Since all-comer histology sarcoma trials are complicated by the underlying subpopulation heterogeneity, many nomograms have been developed to support appropriate disease management. Over 10 nomograms have been developed to evaluate and predict survival probabilities, including specific nomograms for histology and site-of-origin 9–14. The Memorial Sloan Kettering Predictive Nomogram (MSKPN) was based on a multivariate Cox model stratified by histological grade and predicts 4-, 8-, and 12-year probability of sarcoma-specific death 15,16. The MSKPN has been validated by at least three external populations with a concordance index of 0.67–0.76 (0.73 average) 17,18.

Due to the toxicity of AC, we prospectively use MSKPN in our decision to use AC. Patients with a ≥30% 4-year postoperative probability of sarcoma-specific death were defined as high risk and were more likely to be offered AC. We hypothesized that AC (specifically I/Epi) given to high-risk patients would improve patient survival and delay recurrence. As such, we have conducted a retrospective study on STS patients seen at Washington University to investigate this hypothesis.

## Methods

### Patients

**Figure 1:**
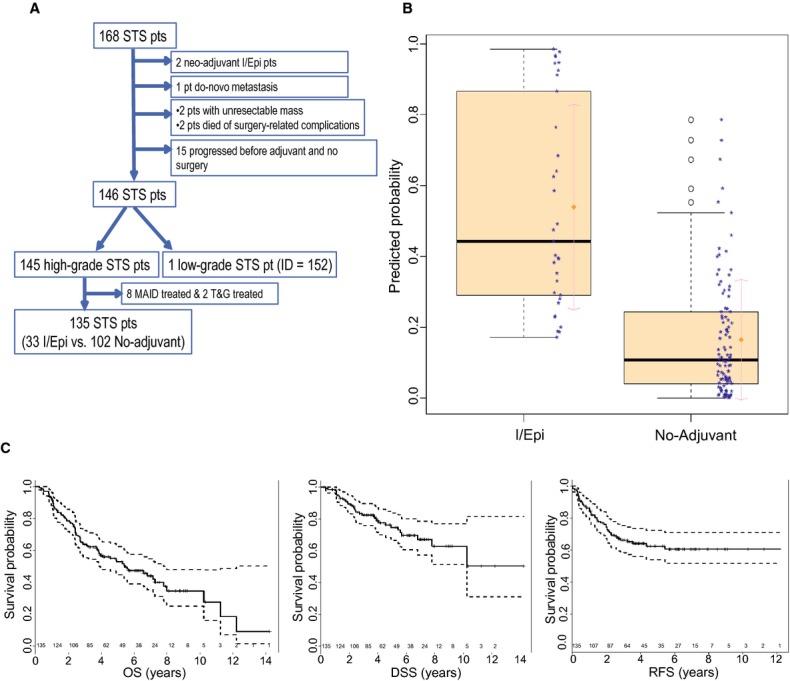
(A) Consort Diagram (patient exclusion conditions are noted). (B) Boxplot of predicted probability of receiving I/Epi from the final logistic regression model for treatment assignment. (C) The overall KM curves of OS, RFS and DSS in the original cohort with 95% confidence intervals (dashed lines) and the number of patients at risk at years 1∼15.

With Institutional Review Board approval, we reviewed the charts of 168 STS patients who were seen by Medical Oncology at Washington University between 15 February 1996 and 6 February 2010. The most recent follow-up date was 20 May 2013. Patient selection flowchart is illustrated in Figure 1A. Patients who progressed before adjuvant treatment, had de novo metastatic disease, or had died from surgical complications were excluded. In 145 high-grade STS patients, 43 received AC (33 I/Epi, 8 doxorubicin/ifosfamide/dacarbazine, 2 taxotere/gemcitibine), while 102 received no adjuvant treatment. We focused on analyzing the 33 patients with adjuvant I/Epi and the 102 patients who had not received AC. For I/Epi AC, all patients were given Ifosfamide was given at 1800 mg/m^2^ over 5 days with epirubicin given at 60 mg/m^2^ on the first 2 days of a 3-week cycle. Patients receiving I/Epi were either chosen for treatment based on a MSKPN risk assessment of death of >30% at 4 years or were seen in second opinion where this decision was independently made.

### Statistical analysis

Categorical variables were summarized as counts and percentages, and between variable associations were examined by Fisher's exact test or *X*^2^ test. Continuous variables were summarized by mean and interquartile range and compared by two-sample *t*-test. OS and disease-specific survival (DSS) were defined as the time interval between date of surgery to date of death or latest follow-up date (20 May 2013). STS disease-specific death, the event indicator for DSS, was defined if a patient's death was preceded by a recurrence or a patient died without a recurrence but with evidence of disease progression. Recurrence was defined as local or distant tumor after 6-month post operation disease free period. RFS was defined as from date of surgery to date of recurrence, date of death, or latest follow-up. The Kaplan–Meier (KM) product limit method was used to estimate empirical survival probabilities. Log-rank test was performed to compare survival difference between/among groups and 5-year survival probabilities were estimated with 95% confidence interval (CI).

The analysis objective was to make causal inference on the effect of AC on survival endpoints. Due to the limitation of a retrospective study where treatment allocation was not randomized, it was impossible to determine whether differences observed in endpoints were due to treatment. As an alternative, the propensity score method 19 was adopted to estimate the likelihood of receiving AC through logistic regression modeling. When subjects have similar propensity scores, observed covariates are automatically controlled and difference in endpoints is attributable to treatment, not observed covariates.

To calculate the propensity scores, the Firth logistic regression model 20, which handles the issues of separability, small sample sizes, and bias of the parameter estimates and estimates model parameters by penalized likelihood method was fit on treatment to derive a full model that included all available covariates. The backward selection method was applied to determine the final logistic regression model for treatment selection where all covariates with a resulting *P*-value ≤0.2 were retained. Odds ratios (OR) and 95% CIs from the logistic regression model were reported on adjusted influence of variables. The propensity scores (the probability of receiving an assigned treatment) were predicted from the final logistic regression model and the weights on subjects were calculated as inverse to the propensity scores.

We evaluated the causal effect of AC on the survival endpoints using the KM method and Cox proportional hazard model with adjustment for the estimated propensity scores using the propensity score inverse probability of treatment weighting (IPTW) approach 19, where each subject is weighted not by conventional equal weight of 1 but by the propensity score IPTW. The proportional hazard assumption was tested for validity of Cox proportional hazard model. In logistic regression models and Cox models, age at diagnosis was considered in its original scale, and after a cubic spline transformation, only results in the original scale were reported since both gave similar results. To provide a *P*-value for the categorical variable histology, the likelihood ratio test was conducted comparing the full (logistic regression or Cox) model against the model leaving histology out. All tests were two-sided, significance was claimed at the 5% level. The statistical computing software R (version 2.15.2; R Foundation for Statistical Computing, Vienna, Austria) 21 was used for statistical analyses. The R package “logistf” 22 was used for the Firth logistic regression and “rms” 23 and “survival” 24 software were used for survival analysis.

## Results

### Patient population

The demographic and clinical/pathological characteristics of the 135 patients, overall and by treatment, are summarized in Table 1. The average age of the entire cohort was 55.4(range: 16–89) years. Overall, gender distribution was balanced (54.1% male patients vs. 45.9% female patients, one-sample proportion test *P* = 0.39). The median tumor size was 7.5 cm with an interquartile range of 5.1–12.4. Overall, 120 (89%) patients had tumors at a deep anatomic depth with the most common tumor site being lower extremity (39%).

**Table 1:** Summary of patient characteristics overall and by treatment

		All patients (*N* = 135)		I/Epi (*N* = 33)		No-adjuvant (*N* = 102)		
					
Variable	Levels	Count	%	Count	%	Count	%	*P*-value
Age at diagnosis	Mean (IQR)	55 (46∼67)		46 (34∼58)		58 (50∼68)		0.00030
Tumor size	≤5	34	25	6	18	28	27	0.53
	5∼10	52	39	15	45	37	36	
	>10	49	36	12	36	37	36	
Depth	Deep	120	89	33	100	87	85	0.022
	Superficial	15	11	0	0	15	15	
Site	Lower extremity	53	39	17	52	36	35	0.50
	Retro-intra-abdominal	27	20	7	21	20	20	
	Head and Neck	5	3.7	1	3.0	4	4.0	
	Thoracic-or-trunk	20	15	2	6.0	18	18	
	Upper-extremity	13	10	3	9.1	10	10	
	Visceral	17	13	3	9.1	14	14	
Histology	Leiomyosarcoma	33	24	6	18	27	26	1.3E-05
	Liposarcoma	22	16	7	21	15	15	
	Undifferentiated pleomorphic sarcoma (ups)	42	31	6	18	36	35	
	Mpnst	13	10	7	21	6	5.9	
	Other	15	11	0	0	15	15	
	Synovial	8	6.0	7	21	1	0.98	
	Fibrosarcoma	2	1.5	0	0	2	2.0	
Gender	F	62	46	11	33	51	50	0.11
	M	73	54	22	67	51	50	
Race	AA	18	13	3	9.1	15	15	0.55
	ASA	3	2.2	0	0	3	2.9	
	C	114	84	30	91	84	82	
Tumor stage	T1	22	16	6	18	16	16	0.79
	T2	113	84	27	81	86	84	
Stage	I/II	27	20	5	15	22	22	0.62
	III	108	80	28	85	80	78	
Margin	−	67	50	15	45	52	51	0.80
	+	47	35	12	36	35	34	
	<10 mm	21	16	6	18	15	15	
Adjuvant radiation	N	50	37	5	15	45	44	0.0033
	Y	85	63	28	85	57	56	
MSKCC risk	Low	63	47	15	45	48	47	1
	High	72	53	18	55	54	53	
Death	Alive	60	44	26	79	34	33	5.8E-06
	Death	75	56	7	21	68	67	
Recurrence	No	86	66	30	94	56	57	7.2E-05
	Yes	45	34	2	6.0	43	43	
DSS	Alive	99	75	31	94	68	69	2.6E-03
	Death	33	25	2	6.0	31	31	

*P*-values were derived comparing a variable's difference between treatment groups. (Fisher's exact test was used for all except for age (Student's *t-*test)).

Seventy-five (56%) patients died while 60 patients were alive at most recent follow-up. Forty-five patients (34%) experienced disease recurrence and 32 (71%) died thereafter. Thirty-three patients were deemed as dead from disease. The median follow-up time was 3.9 years (range: 0.23–14.22 years) and 5.76 years (range: 2.97–14.22 years) among all patients and the survivors, respectively. The KM curves of OS, RFS, DSS with 95% CI and total number of patients at risk for the whole cohort are illustrated (Fig. 1C), from which the median OS was estimated to be 5.42 years (95% CI: 3.92–7.81) while median RFS and DSS were not reached. For patients who received no AC, the median OS, RFS, and DSS were estimated to be 4.05 years (95% CI: 2.75–6.58), 5.43 (95% CI: 2.29-Inf), and 10.23 years (95% CI: 6.79-Inf), respectively.

### MSKPN predicted risk

We followed the MSKPN 25 definitions of tumor characteristics to categorize variables used in the nomogram. The demographic and tumor information were input into the online calculator (http://nomograms.mskcc.org/Sarcoma/PostSurgery.aspx) 15 and the 4-year postoperative probability of sarcoma-specific death predicted. The median 4-year postoperative probability of sarcoma-specific death in the cohort was 0.31 (range: 0.07–0.8). Seventy-two patients (53%) were deemed high risk by the 0.3 cutoff. Using the 0.3 cutoff for risk assessment by MSKPN risk was not significantly associated with any survival endpoint in the whole cohort; neither was the non-dichotomized predicted 4-year death probability (HR, 95% CI, Wald test *P*: 2.61, 0.69–9.96, *P* = 0.16 for OS; 2.41, 0.44–13.38, *P* = 0.31 for RFS; and 3.17, 0.44–23.15, *P* = 0.25 for DSS). When evaluated among the non AC patients, the KM curves showed a trend for better OS in the MSKPN low-risk patients (log rank test *P* = 0.085) and the median survival time was estimated to be 6.58 years (95% CI: 3.13-Inf), and 3.06 (95% CI: 2.33–5.42), low- and high-risk patients, respectively.

The individual variables incorporated in MSKPN were prognostic of survival. Evaluated among non-adjuvant treated patients only, age is significantly associated with OS with a HR of 1.03 (95% CI: 1.01–1.04, *P* = 0.0014) but not with RFS or DSS (HR = 1.01, 0.99–1.02, *P* = 0.57 and 1.02, 0.99–1.04, *P* = 0.20, respectively); histology was found to be prognostic for OS and RFS (log-rank test *P* = 0.009 and 0.004, respectively), tumor size was prognostic for RFS (log-rank test *P* = 0.034).

### Choice of I/Epi

Since ifosfamide is rarely recommended for patients older than 65 years in our practice, the choice of chemotherapy was highly associated with age (Table 1). Patients receiving I/Epi were 12.51 years younger than non-adjuvant-treated patients (mean = 45.91 vs. 58.41, 95% CI on the mean difference: 6.04–18.96, *t*-test *P* = 0.0003). Tumor depth and histology were also significantly associated with treatment (Table 1). Among histology types, seven of eight synovial tumors received I/Epi, while the majority of other histology subtypes received no chemotherapy (Fisher's exact test *P* = 1.31E-05). Other patient or tumor characteristics were not significantly associated with treatment (Table 1). The median MSKPN predicted 4-year postoperative probability of sarcoma-specific death was 0.30 (range 0.15–0.73) in I/Epi-treated versus 0.31 (range 0.07–0.8) in non AC patients, not significant (Wilcoxon rank sum test *P* = 0.63). By the 0.3 cutoff, 18 (25%) of 72 high-risk patients and 15 (24%) of 63 low-risk patients received I/Epi (Fisher's exact test *P* = 1). Thus, the choice of I/Epi over no AC was not significantly associated with MSKPN-predicted risk reflecting the number of second opinion referrals at Washington University. The age difference between I/Epi-treated and non-adjuvant-treated patients was significant even after stratification by MSKPN. The mean age of I/Epi-treated patients was 48.5 years among high-risk patients, (62.83 in non-treated high-risk patients, 95% CI on the mean difference = 5.13–23.54; *t*-test *P* = 0.004) and 42.8 years among low-risk patients (53.44 years in non AC low-risk patients, 95% CI on the mean difference = 1.30–19.97; *t*-test *P* = 0.035).

Patients who received I/Epi tended to receive radiation. Twenty-eight (84.85%) of the 33 I/Epi-treated patients received radiation, higher than those in the non-treated patients (55.89%, Fisher's exact test *P* = 0.0033, Table 1). Patients who had radiation showed better survivals in the whole cohort (log rank test *P* = 0.043, 0.034, 0.073 for OS, RFS, and DSS, respectively). Since radiation did not demonstrate an overall survival benefit in the non-adjuvant-treated patients (log-rank test *P* = 0.355, 0.287, 0.394 for OS, RFS, and DSS, respectively), the overall survival benefit in the high-risk patients can likely be attributed to I/Epi treatment by using IPTW modeling.

The propensity score methods 19 were applied to model the likelihood of receiving I/Epi using multivariate Firth logistic regression model 20. Initially, a full model including all available covariates (age, radiation, histology, MSKPN binary risk, tumor size, race, gender, site, depth, tumor stage, and margin) was fit followed by backward selection procedure to arrive at a final logistic regression model with the four covariates: age, radiation, histology, and MSKPN binary risk (Table 2). Histology was a strong influential factor (likelihood ratio test *P* = 0.0011), but treatment administration was variable by histological subtype. High-risk patients had a higher likelihood of receiving chemotherapy, although not statistically significant at the 5% level reflecting the number of patients seen in second opinion. The propensity scores, that is the predicted probability of receiving I/Epi was calculated from the final logistic model and boxplot on the scores (Fig. 1B) by actual treatment assignment indicated that the model well captured the likelihood of receiving I/Epi.

**Table 2:** Multivariate logistic regression model for likelihood of receiving I/Epi

Variable	Odds ratio (95% CI)	*P*-value
Age at diagnosis	0.94 (0.9–0.97)	0.0004
Radiation (yes vs. no)	11 (2.76–60.63)	0.0002
Histology		0.0011
Liposarcoma versus leiomyosarcoma	3.8 (0.91–17.78)	0.068
Ups versus leiomyosarcoma	0.52 (0.13–2.07)	0.35
Mpnst versus leiomyosarcoma	4.4 (0.85–25.05)	0.078
Synovial versus leiomyosarcoma	22 (2.18–410.58)	0.0069
Fibrosarcoma versus leiomyosarcoma	0.98 (0.01–26.63)	0.99
Other versus leiomyosarcoma	0.26 (0–2.94)	0.32
Risk (high vs. low)	2.4 (0.76–8.55)	0.14

### Effect of chemotherapy in STS patients

**Figure 2:**
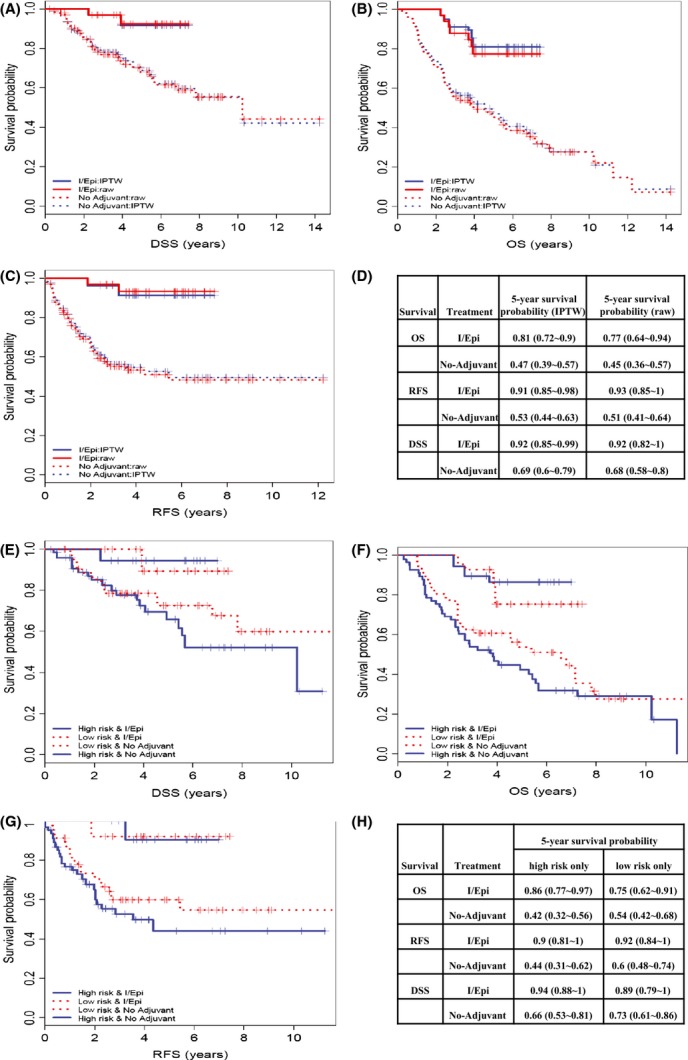
(A–D) The KM curves of DSS (A), OS (B), and RFS (C) by treatment (IPTW: solid lines; No-adjuvant: dotted lines) in the raw cohort (red lines) and the IPTW cohort (blue lines) and the associated 5-year survival probability estimations with 95% CI in parenthesis (D); (E–H): The KM curves of DSS (E), OS (F), RFS (G) by high/low MSKPN risk and treatment combinations using the IPTW cohort and 5-year survival probability estimations with 95% CI in parenthesis (H).

We evaluated the influence of treatment (I/Epi or no AC) on survival using a propensity score IPTW method in the context of survival analysis, where the weight on patients were calculated as inverse to the predicted probabilities of receiving their assigned treatments to reduce potential selection bias. Plotted in Figure 2 are the KM curves and the 5-year absolute survival probability by treatment estimated on the IPTW cohort in comparison to the original cohort where all subjects had an equal weight of 1. Chemotherapy was associated with an improved 5-year survival probabilities from 47% to 81%, 53% to 91%, and 69% to 92% for OS, RFS, and DSS, respectively, with absolute differences ranging from 23% to 38%. By Cox regression modeling, hazards of dying (general or disease-specific) and relapsing were dramatically higher when patients were not treated with chemotherapy (Table 3). The HRs estimated on the IPTW cohort were slightly smaller compared to the estimates on original cohort for RFS and DSS but increased for OS. The OS benefit demonstrated in this retrospective study is much higher than the Italian study, which can be attributed to risk assessment, inclusion of sites other than extremity, retrospective approach, and/or small sample size.

**Table 3:** Univariate Cox regression model evaluating treatment effect in the IPTW cohort, overall and then by MSKPN risk

Survival	All patients (*N* = 135)		High risk patients (*N* = 72)		Low risk patients (*N* = 63)	
		
	HR (95% CI)	*P*-value	HR (95% CI)	*P*-value	HR (95% CI)	*P*-value
OS	4.43 (2.53–7.76)	1.9E-07	6.94 (2.96–16.29)	8.4E-06	2.91 (1.37–6.18)	0.0054
RFS	8.02 (3.57–18.05)	4.8E-07	9.54 (3.16–28.77)	6.2E-05	6.51 (1.97–21.55)	0.0022
DSS	5.57 (2.29–13.53)	0.00015	8.2 (2.26–29.68)	0.0013	3.82 (1.11–13.13)	0.033

Hazard ratios (HRs) refer to non AC versus I/Epi.

When treatment was investigated under stratification of MSKPN risk, both high- and low-risk patients benefited from adjuvant I/Epi, however, high-risk patients demonstrated a much greater benefit. The resulting hazard ratios (I/Epi vs. no-AC) were much higher in high-risk patients for all the survival outcomes (Table 3), although the differences were not significant (Wald test *P* = 0.16, 0.66, and 0.44 for OS, RFS, and DSS, respectively). Examining the 5-year survival probabilities, significant benefit for I/Epi was found in both high- and low-risk groups (Fig. 2E–H). The need for stratification of patients into high-risk and low-risk groups may begin to explain the difficulty in seeing overall survival differences when all-comers are used in clinical trials.

To evaluate whether treatment improved survival when considering the influential clinico-pathological variables and adjusting for the propensity scores to reduce treatment selection bias, we applied multivariate Cox models on the propensity score IPTW cohort for all survival endpoints (Table 4). The adjusted HR of treatment (untreated vs. I/Epi) was 4.18 (95% CI: 2.22–7.90), 8.96 (95% CI: 3.85–20.83), and 5.42 (95% CI: 2.09∼14.06) for OS, RFS, and DSS (Table 4), respectively, indicating a strong independent influence of adjuvant treatment with I/Epi on survival in addition to all other variables in the model. Radiation therapy and depth did not show significant HRs when treatment and other variables were included, while age, histology, and tumor size were significantly associated with at least one survival endpoint.

**Table 4:** Multivariate Cox regression model in the IPTW cohort

Variable	OS		RFS		DSS	
		
	Hazard ratio (95% CI)	*P*-value	Hazard ratio (95% CI)	*P*-value	Hazard ratio (95% CI)	*P*-value
Treatment (no-adjuvant vs. I/Epi)	4.18 (2.22–7.9)	1.0E-05	8.96 (3.85–20.83)	3.5E-07	5.42 (2.09–14.06)	0.0005
Age at diagnosis	1.02 (1.01–1.04)	0.008	1.0 (0.98–1.02)	0.86	1.01 (0.99–1.04)	0.30
Histology		1.1E-06		0.0003		0.078
Liposarcoma versus leiomyosarcoma	0.96 (0.49–1.88)	0.90	0.17 (0.07–0.42)	9.1E-05	0.32 (0.11–0.91)	0.032
Mfh versus leiomyosarcoma	1.4 (0.76–2.59)	0.28	0.46 (0.24–0.87)	0.018	0.87 (0.38–2.02)	0.75
Mpnst versus leiomyosarcoma	0.27 (0.08–0.90)	0.034	0.21 (0.07–0.6)	0.0035	0.39 (0.11–1.34)	0.14
Other versus leiomyosarcoma	2.82 (1.32–6.02)	0.0075	0.19 (0.04–0.79)	0.022	0.6 (0.13–2.66)	0.50
Synovial versus leiomyosarcoma	7.91 (2.94–21.24)	4.1E-05	0.0009 (0–Inf)	0.56	2.32 (0.48–11.26)	0.30
Fibrosarcoma versus leiomyosarcoma	9.47 (2.05–43.80)	0.0040	1.44 (0.17–11.94)	0.73	3.73 (0.38–36.59)	0.26
Radiation (yes vs. no)	0.92 (0.58–1.47)	0.74	0.72 (0.4–1.29)	0.27	0.74 (0.38–1.43)	0.37
Risk (high vs. low)	1.09 (0.6–1.97)	0.78	0.74 (0.36–1.51)	0.41	0.86 (0.36–2.09)	0.74
Depth (superficial vs. deep)	0.96 (0.42–2.17)	0.92	0.62 (0.18–2.16)	0.45	1.09 (0.29–4.09)	0.90
Tumor size		0.16		0.11		0.067
5∼10 versus ≤5	1.36 (0.65–2.84)	0.42	2 (0.77–5.23)	0.16	1.52 (0.47–4.94)	0.49
>10 versus ≤5	1.9 (0.91–3.98)	0.087	2.69 (1.05–6.92)	0.040	3.01 (0.95–9.59)	0.062

## Discussion

AC for resectable STS remains controversial and the subject of adamant debate. Given Washington University's use of adjuvant I/Epi, we undertook a retrospective analysis of 33 I/Epi-treated and 102 non-adjuvant-treated patients. Overall, we found I/Epi corresponded to significant improved DSS, OS, and RFS with greatest benefit among patients at high risk of recurrence.

Our analysis revealed no significant difference in the median MSKPN-predicted risk between adjuvant I/Epi-treated versus non-treated patients. However, 46% of patients receiving I/Epi were of low risk. This is a result of patients captured in the data set that were seen as second opinions. To remove potential influence of I/Epi treatment, we independently analyzed all 102 patients who received no-AC and identified a trend of improved OS in MSKPN predicted low- versus high-risk patients (log-rank test *P* = 0.085). Among the MSKPN individual prognostic variables 15, histology and tumor size showed significant prognosis effect in our data. These findings may have been influenced by including only high-grade STS, a relatively small sample size, and using a 4-year predicted probability of sarcoma-specific death.

To evaluate whether adjuvant I/Epi treatment improved survival while reducing potential bias from treatment selection, we implemented the propensity score IPTW approach in the context of KM analysis and Cox models to alleviate the limitations of this retrospective study. When we compared the 5-year survival probability between I/Epi versus non-adjuvant-treated patients, we found a difference of 28%, 44%, and 46% for DSS, OS, and RFS in high-risk patients, and a smaller difference in low-risk patients.

Adjuvant radiotherapy is commonly used with close or positive margins. In our data set, radiation therapy was given to 85 patients (85% I/Epi-treated versus 56% untreated) with high-grade STS with margin status (35 positive margins, 18 < 1 cm, 32 > 1 cm) and within a variable set of histologies. In prospective studies high-grade STS patients receiving postoperative radiotherapy demonstrated a lower risk of local recurrence 26. In our data set, radiation was associated with improved RFS and OS, IPTW modeling allows us to attribute the RFS and OS to AC in high-risk patients, as patients who received radiation were more likely to receive AC. Prior trials of AC in STS have ranged in size from approximately 43 to 245 patients (Mean = 100) 7,27. Interestingly, the 5-year OS probability for the non AC group was 0.46 in the Italian study 7, similar to our population (0.47). In our I/Epi-treated group, the OS probability was 0.81 versus 0.66 in the Italian study 7. However, the Italian study selected only high-grade extremity STS, while our analysis analyzed all primary locations.

Despite the dominance of doxorubicin-based AC in other studies, these data support epirubicin as a viable alternative, as originally demonstrated by Frustaci et al. 7. Despite our attempts to mitigate selection bias through a propensity IPTW method, these data are hypothesis generating only. The large number of STS histologies and variation in the use of neoadjuvant or AC between centers suggests revisiting the use of epirubicin as part of adjuvant therapy. This could be achieved through a randomized trial through a cooperative group or consortium, where stratification by histology, biomarker, or genetic signature could be performed. Without further primary data, the field will be left with unanswered questions regarding the best agents for neoadjuvant or adjuvant therapy and who, if anyone, would benefit most. Although this trial will be costly and difficult to fund, the importance of AC for sarcoma still needs to be addressed.

Finally, epirubicin dosing has not been established in sarcoma where higher dosing can be used with less risk of cardiotoxicity 28. Although Lopez et al. showed a higher response rate at higher dosing of epirubicin 29, the AC epirubicin total dosing of 600 mg/m^2^ 7 compares to the breast cancer clinical trial literature, where total cumulative dosage is between 360 mg/m^2^ and 800 mg/m^2^ 30. The opportunity to increase the total cumulative dosage above 600 mg/m^2^ 7 may also be warranted in a clinical trial setting to try to improve outcomes.

In summary, within the limits of our retrospective analysis, I/Epi-treated patients regardless of their MSKPN risk of relapse have a significantly greater OS, RFS, and DSS compared to those not receiving AC. Therefore, randomized, appropriately powered, prospective clinical investigations are warranted to further investigate if I/Epi is superior to I/Doxorubicin for AC in STS.
